# Human Cytomegalovirus Associated Neuropathies: A Comprehensive Review From Pathophysiology to Clinical and Therapeutic Considerations

**DOI:** 10.1111/jns.70087

**Published:** 2025-12-08

**Authors:** Naomi Behanan, Sarab Mohamed, Rhona Chen, Gloria Mak, Jian‐Qiang Lu

**Affiliations:** ^1^ Department of Pathology and Molecular Medicine McMaster University Hamilton Ontario Canada; ^2^ Health Sciences McMaster University Hamilton Ontario Canada; ^3^ Department of Neurology/Medicine The University of Alberta Edmonton Alberta Canada

**Keywords:** autoimmunity, cytomegalovirus, nerve infection, neuropathies, pathogenesis

## Abstract

Human cytomegalovirus (HCMV) is a neurotropic, double‐stranded DNA virus from the Herpesviridae family. It has a large genome, infects the majority of populations, and typically causes asymptomatic infections in healthy individuals. After the initial infection with established lifelong latency, HCMV can reactivate and cause disorders including neuropathies. Besides the infections typically in immunocompromised patients, HCMV may also trigger autoimmunity leading to tissue injury and associated pathologies. HCMV‐associated neuropathies are a pathogenically heterogeneous group of peripheral nervous system (PNS) disorders that include direct HCMV infection of the nerve(s), as well as non‐infectious associated neuropathies such as axonal or degenerative, vasculitic/ischemic or necrotizing, inflammatory demyelinating, and immune‐mediated forms. Congenital HCMV‐associated neuropathies primarily involve the cochlear/auditory and optic nerves with somewhat distinct pathogenic mechanisms compared with their postnatal counterparts, largely due to the immaturity of the fetal immune system. This article reviews the pathophysiology of PNS involvement in HCMV infection, followed by congenital HCMV‐associated neuropathies with a case demonstration, and various postnatal HCMV‐associated neuropathies, including a detailed review of HCMV‐associated optic neuropathies, from pathogenic mechanisms to clinical and therapeutic implications. As the PNS has a few immune protective mechanisms against pathogens, direct HCMV infection of nerves is rare and occurs only in immunocompromised patients; most HCMV‐associated neuropathies are secondary with multiple pathogenic mechanisms including varying degrees of autoimmunity. While the clinical manifestations of HCMV‐associated neuropathies are variable, their treatment is typically empirical and case‐based, focusing on antiviral therapy often combined with immunomodulatory approaches. Prompt and appropriate management can improve outcomes of HCMV‐associated neuropathies.

## 1 Introduction

Human cytomegalovirus (HCMV) is a member of the Herpesviridae family, including eight known human herpesviruses. It is a large, enveloped, double‐stranded DNA virus with a broad cell tropism and a complex genome, significantly larger than most human viruses. HCMV infects 60%–90% of global populations, with the majority of infections being asymptomatic in healthy individuals [1, 2]. Like all human herpesviruses, HCMV can establish lifelong latency once infected, and later, reactivate under conditions of immunosuppression or physiological stress. It is neurotropic and capable of causing neurological disorders including peripheral neuropathies. HCMV can infect the peripheral nerves by directly invading the nerve endings in the tissue, as these nerves are in direct contact with various tissues/organs that are more susceptible to HCMV [3]. Nevertheless, given the peripheral nervous system (PNS) protective mechanisms particularly the blood‐nerve barrier (BNB), HCMV direct infection of the PNS is rare and primarily occurs in immunocompromised individuals [4, 5]. In contrast, hematogenous spread of HCMV infection is relatively common as HCMV‐infected cells of the circulatory system can carry the infection through the BNB into the PNS. Understanding its biology is essential for contextualizing its role in the pathogenesis and clinical manifestations of PNS disorders.

HCMV infection is a common systemic opportunistic infection in patients with immunocompromised conditions, such as acquired immune deficiency syndrome (AIDS) including human immunodeficiency virus (HIV) infection, and is occasionally seen in immunocompetent individuals [1, 2, 3, 4, 5, 6, 7]. Moreover, congenital HCMV (cHCMV) infection is the most common congenital infection and the leading cause of non‐genetic sensorineural hearing loss in children. It is also a less common cause of other neurological disorders, including visual abnormalities [8, 9, 10]. While HCMV infection can affect multiple systems, HCMV has a particular predilection for the central nervous system (CNS), retina, and PNS [1, 6, 11]. Given this predilection, it may be more informative to explore HCMV infection and its associated pathologies in specific systems, such as the PNS, to better understand its pathogenesis and clinical manifestations.

PNS HCMV infection and its associated pathologies can be anatomically divided into cranial and spinal neuropathies. Common HCMV‐associated cranial neuropathies include optic neuropathies and auditory or vestibulocochlear neuropathies, while involvement of other cranial nerves by HCMV is extremely rare. HCMV‐associated spinal neuropathies mainly manifest as Guillain–Barré syndrome (GBS; also known as acute inflammatory demyelinating polyradiculoneuropathy, AIDP), chronic inflammatory demyelinating polyneuropathy (CIDP), HCMV‐associated polyradiculomyelopathy (originally known as acute lumbosacral polyradiculopathy in AIDS patients), multiple mononeuropathy (originally reported as multifocal neuropathy in association with extraneurologic HCMV infection), and/or distal peripheral neuropathy [7, 12, 13, 14]. The pathogenic mechanisms of these HCMV‐associated neuropathies are diverse and include direct HCMV infection, HCMV‐induced autoimmunity, secondary degeneration, and necrosis/infarction (Table 1). However, cHCMV‐associated neuropathies may be pathogenically different from their postnatal counterparts, given the fetal developing immune system. This article reviews the PNS pathophysiology in HCMV infection followed by cHCMV‐associated neuropathies with a case demonstration, and various non‐congenital HCMV‐associated neuropathies, including details of HCMV‐associated optic neuropathies, from pathogenic mechanisms to clinical and therapeutic implications.

**TABLE 1:** HCMV‐associated neuropathies.

HCMV direct infection versus Immune‐mediated neuropathies	
HCMV direct infection, infectious neuropathy, caused by HCMV direct invasion into PNS tissue Typically in immunocompromised or congenital conditions Usually contiguous with the retinal/ocular or CNS HCMV infection Typically presenting as a necrotizing neuropathy Possibly associated with one or more of the following: Vasculitic/ischemic neuropathies Axonal or degenerative neuropathies including Wallerian degeneration Mixed or unclassified neuropathies	Immune‐mediated neuropathies, associated with systemic HCMV infection causing immune‐mediated attack on PNS tissue With or without autoantibodies (anti‐AQP4, anti‐MOG, anti‐MAG/SGPG, anti‐GalNAc‐GD1, anti‐glycolipid, or anti‐ganglioside; see Table S1) In immunocompromised or immunocompetent individuals Presenting as one or more of the following: Inflammatory demyelinating neuropathies (most commonly, AIDP/GBS, CIDP)—axonal or degenerative neuropathies including Wallerian degeneration Vasculitic/ischemic neuropathies Mixed or unclassified neuropathies

Abbreviations: AIDP, acute inflammatory demyelinating polyneuropathy (or GBS, Guillain–Barré syndrome); CIDP, chronic inflammatory demyelinating polyneuropathy; CNS, central nervous system; HCMV, human cytomegalovirus; PNS, peripheral nervous system.

## 2 Pathophysiology of PNS in HCMV Infection

HCMV infections are commonly asymptomatic or mildly symptomatic when there is immune competence, as they are controlled by a vigorous immune response [2]. The PNS has traditionally been considered an immunologically privileged site, although not as stringently as the CNS [15]. It has unique protective mechanisms against pathogenic attacks. These mechanisms include the BNB restricting access of circulating pathogens, and immune surveillance patrolled by activated T and B lymphocytes, as well as macrophages and Schwann cells functioning as antigen‐presenting cells, contributing to the local immune network [15, 16] Despite these protective mechanisms, the PNS is not fully immune to viral invasion or immune‐mediated injury, particularly in immunocompromised hosts. In the setting of an infection, molecular crosstalk between the PNS and immune system can occur, thereby affecting barrier homeostasis, host defense, and function [17].

It is important to distinguish between congenital and postnatal HCMV infections when considering PNS involvement. Congenital HCMV infection is specifically described later in this article. Postnatal HCMV infection of the PNS occurs almost exclusively in immunosuppressed individuals [4, 5]. In this setting, reactivation of latent virus or reinfection can lead to direct and/or immune‐mediated nerve injury (Table 1). HCMV can replicate in various cell types, such as endothelial cells, and myeloid‐derived cells, facilitating viral spread to the other tissues and cell types including neurons [1]. The host cells are modulated by HCMV interfering with signaling pathways for innate or acquired immune responses. Once primary infection occurs, HCMV spreads through the bloodstream to various organs, which can become latent in circulating monocytes and bone marrow cells [1, 18]. HCMV‐infected monocytes are capable of recirculating to the bone marrow while harboring the virus, and differentiating into patrolling monocytes that can be activated to differentiate into macrophages. Macrophages and their progenitor cells can recognize common non‐self‐pathogen‐associated molecular patterns and play a pivotal role in the systemic HCMV dissemination and latency [1, 18, 19].

T cells and B cells also play important roles in HCMV infection, as CD4+ and CD8+ T cells regulate the balance between persistent and latent HCMV infection [1, 20]. HCMV leads to an initial response dominated by CD4+ T cells producing T‐helper 1 type cytokines. The later immune response is primarily driven by CMV‐specific cytotoxic CD8+ T cells targeting several viral antigens, such as the immediate‐early protein IE‐1 and the tegument phosphoprotein pp65 [1, 20, 21]. HCMV infection leads to the infiltration and accumulation of antiviral CD8+ T cells in some particular organs/tissues such as the brain and eye, and promotes the development of tissue‐resident memory T cells, which can drive long‐lasting inflammation and cause latent infection [22, 23]. HCMV reactivation may be triggered by factors associated with the inflammatory response, leading to the reinitiation of viral replication and subsequent dissemination to various organs and tissues including the PNS [1, 20, 21].

The PNS may be affected not only by direct HCMV infection, but also by HCMV‐induced autoimmunity potentially through molecular mimicry [13, 24, 25]. HCMV can employ molecular mimicry as a strategy for immune evasion and induce autoimmune responses [26]. In the process of molecular mimicry triggered by an infection, autoreactive T cells recognize a specific autoantigen presented by major histocompatibility complex class II molecules and the simultaneous delivery of costimulatory signals on the cell surface of antigen‐presenting cells in the systemic immune compartment. These activated T cells can cross the BNB to enter the PNS. Within the PNS, T cells activate macrophages that enhance phagocytic activity, production of cytokines and the release of toxic mediators including nitric oxide, matrix metalloproteinases, and proinflammatory cytokines such as tumour necrosis factor‐alpha (TNF‐α) or interferon‐gamma (IFN‐g). Autoimmune responses targeting the PNS structures are initiated by the presence of autoantibodies that may either originate from extra‐PNS tissue or be locally produced by B cells. Autoantibodies, such as those against gangliosides including GM1 and GM2, have been found in autoimmune neuropathies such as GBS and CIDP, which are commonly associated with infections including HCMV [1, 15, 27, 28, 29]. These autoantibodies contribute to the immune‐mediated process of demyelination and axonal damage. This process involves immune cells including T cells, B cells, and macrophages that interact with endogenous, partially immune‐competent glial cells, particularly Schwann cells, and contribute to local inflammation [15, 16]. These immune/inflammatory changes are typically observed in post‐infectious immune‐mediated neuropathies such as GBS and CIDP, which are pathologically characterized by immune/inflammatory cell infiltrates associated with demyelination and axonal loss in the affected part of the nerve [1, 15, 24]. A wide range of infectious agents have been implicated in the etiopathogenesis of GBS, including *Campylobacter jejuni* , HCMV, Epstein–Barr virus, varicella‐zoster virus, herpes simplex virus, hepatitis viruses, influenza, HIV, dengue virus and Zika virus [30, 31]. HCMV, in particular, has been associated with GBS through its ability to trigger autoimmunity via molecular mimicry, as described above [24, 25], since antecedent HCMV infection has been found in ~13% of GBS cases [30, 31]. CIDP, while clinically similar in some respects to GBS, differs in its chronic course and pathophysiology. Although its association with a persistent viral infection is not well established [31], there have been a few CIDP cases with detected HCMV suggestive of HCMV reactivation (Table S2).

## 3 Literature Search Strategy and Selection Criteria

We conducted a systematic literature search in PubMed/MEDLINE and Web of Science databases for English‐language articles published from inception to April 2025. The search terms included “cytomegalovirus” or “CMV” and (combined with) “neuropathy”, “nerve”, “peripheral nervous system”, “neuritis”, “optic”, or “papillitis”. While each search yielded a variable number of publications (ranging from dozens to hundreds), we excluded publications that did not align with the scope of this review, such as nonhuman studies. Our review identified cases and studies describing various neuropathies caused by HCMV infection with HCMV identified in the nerve tissue or associated with concurrent or preceding HCMV infection evidenced by detection of the virus in the serum/blood, cerebrospinal fluid (CSF), and/or other non‐nerve tissue. HCMV‐associated optic neuropathies were particularly reviewed in depth given their special pathophysiology and clinical implications.

## 4 Congenital HCMV Infection and Associated Neuropathies

### 4.1 Prevalence

cHCMV infection is the most common congenital infection worldwide [8, 32]. It affects approximately 1% of live births in high‐income countries including the United States, and up to 2% in low‐and middle‐income countries [8, 10]. HCMV can lead to significant morbidity and mortality, especially in neonates. cHCMV is a major cause of neurological damage and the leading non‐genetic cause of sensorineural hearing loss (SNHL), accounting for approximately 25% of all hearing loss in children by 4 years of age [8, 9, 33]. Although cHCMV imposes significant health, social, and economic burdens, it often remains underdiagnosed at birth because most infants are asymptomatic, or show only nonspecific signs that do not raise clinical suspicion [32]. At birth, 10%–15% cHCMV‐infected infants exhibit noticeable symptoms such as petechiae, jaundice, hepatosplenomegaly, microcephaly, and neuropathy. However, 85%–90% of cHCMV‐infected infants are asymptomatic, resulting in many cases remaining undiagnosed in the absence of routine antenatal or neonatal screening programs [8, 32].

HCMV infection during pregnancy may occur as a primary infection, reactivation of a latent infection, or reinfection with a new strain [8, 32]. Pregnant individuals are most commonly infected through contact with the saliva and urine of young children. Primary HCMV infections during pregnancy have a high in utero transmission risk of 30%–35%, whereas non‐primary infections carry a much lower risk of 1.1%–1.7%. While the risk of transmission to the fetus increases later in pregnancy, infections earlier in gestation carry a higher risk of fetal harm [32]. Congenital infections can lead to serious long‐term effects, including permanent hearing and vision loss as well as neurological impairments [34].

### 4.2 Pathogenesis of Congenital HCMV Infection

HCMV can be transmitted to the fetus before birth when the virus crosses the placenta and replicates in various fetal tissues [34]. Timing of maternal infection and maternal immune status largely determine the likelihood of a symptomatic cHCMV infection, while the fetal immune system also contributes to the outcome of cHCMV infection [35]. It is important to note that the fetal immune system is still developing and immature particularly during the first and second trimesters [35], which makes the pathogenic mechanisms of cHCMV infection distinct. HCMV can harm the fetus through multiple complex mechanisms, including direct viral damage, impaired maternal immune control, and disruption of placental function [36]. However, the exact mechanisms by which cHCMV infection causes fetal injury are not well understood [35, 36]. It has been proposed that the virus produces gene products that interfere with key cellular processes such as cell cycle regulation, apoptosis, inflammation, vascular integrity, chromosome stability, immune evasion, and abnormal cell growth [36]. Experimental findings have led to several hypotheses regarding the mechanisms by which HCMV causes fetal injury, including interference with normal development, vascular damage, chromosomal alteration, and placental dysfunction. Despite progress in understanding HCMV's cellular effects, the exact mechanisms of fetal harm remain elusive [36].

### 4.3 Congenital HCMV‐Associated Neuropathies

The common sequelae of cHCMV infection include SNHL that may be caused by cochlear/auditory neuropathy, and visual impairment that may be caused by retinal/ocular disorder and/or optic neuropathy. A better understanding of these long‐term sequelae can facilitate early detection, timely intervention, and implementation of appropriate educational accommodations [9, 10, 33]. In a study of 153,913 newborns with hearing loss, cochlear nerve deficiency accounts for 3.0% of moderate cases and 1.6% of severe to profound cases [37]. The pathogenesis of cHCMV‐associated SNHL has been thought to be from viral cytopathic effects and local inflammatory responses, commonly involving the cochlear nerve. HCMV‐infected cells have been detected in the spiral ganglion and cochlear nerve in some reported cases; inflammatory cell infiltrates, including CD8+ T cells, B cells and macrophages, have been found in these regions [9, 33]. The majority of children with HCMV‐associated SNHL experience progression of hearing impairment during early childhood; additionally, among many asymptomatic cHCMV cases at birth, ~5% develop late‐onset SNHL during early childhood [33]. Therefore, close monitoring of all HCMV‐infected children for SNHL during early childhood is essential to enable prompt intervention, alongside universal newborn HCMV and hearing screening programs.

**FIGURE 1:**
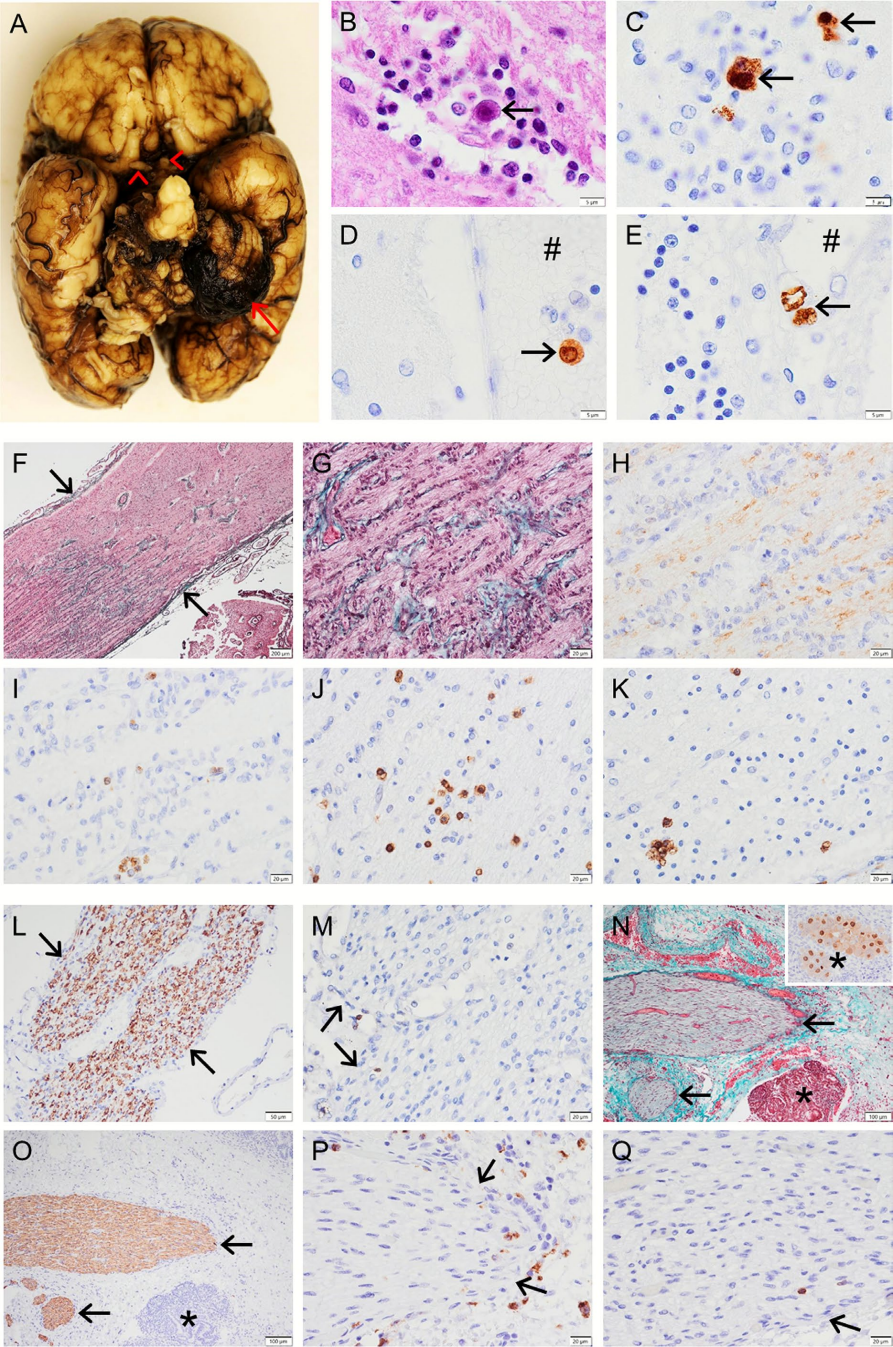
HCMV infection‐associated optic neuropathy. (A) The brain gross examination reveals cerebellar hemorrhage (arrow) and prominent optic nerves (arrowheads). (B–E): Microphotographs of the brain show microglial nodules (B; C) containing large cells with nuclear inclusions (B, arrow) and positive HCMV immunostaining (C, arrows). HCMV+ cells are also found within the blood vessel lumen (D, #) and leptomeninges (E; #, blood vessel), suggestive of HCMV hematogenous dissemination and entry into the CNS. (F–K): Proximal optic nerve demonstrates loss of nerve fibers with variable fibrotic/reactive changes (E, Masson Trichrome staining; F, higher magnification), marked loss of NFP immunostaining for axons (H), and inflammatory cell infiltrates including CD68+ macrophages (I), CD3+ T cells (J) and CD8+ cytotoxic T cells (focally clustered; K). In comparison, the trigeminal nerve is unremarkable with well‐preserved NFP+ axons/fibers (L, arrows) and rare CD3+ T cells at the nerve periphery (M, arrows). The visceral nerves (N, arrows) adjacent to the HCMV‐infected kidney (N, *; inset, HCMV immunostaining+ cells) are also unremarkable with well‐preserved NFP+ axons/fibers (O, arrows), occasional CD68+ macrophages at the nerve periphery despite more in the adjacent tissue (P, arrows), and rare CD3+ T cells at the nerve periphery (Q, arrow). Scale bar: 5 μm (B–E), 200 μm (F), 20 μm (G–M, O–Q), 100 μm (N).

The ocular cHCMV disease accounts for approximately 20% of children with HCMV viremia, in which optic nerve involvement occurs in less than 10% of cases [38]. A prospective study of 237 infants and children with cHCMV infection (confirmed by the urine culture within 3 days of life) examined long‐term ophthalmologic sequelae, revealing that optic nerve atrophy was a leading ophthalmologic finding and a significant cause of severe visual impairment occurring exclusively in symptomatic patients (10.4%) [10]. Moreover, optic nerve atrophy was found to be associated with SNHL, as both were manifestations of cHCMV [10]. Given the aforementioned nerve protective mechanisms, cHCMV infection involving other peripheral nerves (excluding the optic and cochlear/auditory nerves) has not been reported. The present case demonstrates a secondary axonal/degenerative optic neuropathy associated with brain and systemic cHCMV infection, while the other peripheral nerves remain unremarkable (Figure 1). Relevant non‐cHCMV optic neuropathies will be reviewed in detail next.

## 5 Case Vignette of Congenital HCMV‐Associated Optic Neuropathy

A premature hydropic male infant was delivered by emergency C‐section at 28 weeks and 3 days gestation due to minimal fetal heart rate variability. The mother underwent TORCH and other infections screening 16 days prior to emergency C‐section as per pregnancy was complicated by hydrops fetalis, hypertension and anhydramnios. Her screening was negative and she had HCMV IgG positivity with IgM negativity. At birth, the baby required significant resuscitation, which included drainage of pleural and peritoneal fluid via bilateral chest tubes and paracentesis, as well as drain replacement for bilateral pneumothorax and ascites. Despite these resuscitation efforts, he deteriorated and died on day 1 of life. Post‐mortem examination revealed HCMV infection, confirmed by the presence of characteristic viral inclusions and positive HCMV immunostaining in multiple organs including lung, liver, thyroid, kidney and brain. The brain also showed variable meningoencephalitic changes in multiple regions, characterized by parenchymal microglial nodules and inflammatory cell infiltrates including macrophages/microglia and T cells, as well as cerebellar hemorrhage (Figure 1A–E).

The proximal optic nerve (ON) exhibited marked nerve fiber loss with scattered CD68+ macrophages and frequent CD3+ or CD8+ T cells (Figure 1F–K), in the absence of CD4+ T cells or CD20+ B cells. Variable gliotic/reactive changes were also observed, accompanied by abundant S100+ Schwann cells. Compared with the ON, the adjacent optic chiasm and brain tissue showed fewer inflammatory cell infiltrates. HCMV immunostaining was negative in the ON, despite being positive in the brain. Polymerase chain reaction (PCR) testing for HCMV in the ON tissue was also negative. For comparison, we also examined the other cranial nerves, multiple spinal nerve roots and visceral nerves. These non‐optic nerves were all unremarkable, showing well‐preserved nerve fibers and minimal to limited inflammatory cell infiltrates at the nerve periphery, despite frequent HCMV infection in the adjacent tissue and organs (Figure 1L–Q). Our findings in this case suggest HCMV infection‐associated optic neuropathy that is pathologically axonal, degenerative neuropathy with secondary changes including fibrosis and inflammatory cell infiltrates but no evidence of direct HCMV invasion into the ON.

## 6 Postnatal HCMV‐Associated Optic Neuropathies

Table S1 summarizes our present case and 32 previously reported cases of HCMV‐associated optic neuropathies (including optic neuritis; published in English literature). In this summary table, we excluded the previous cases and studies of ocular or retinal HCMV infection lacking convincing involvement of the ON, as well as clinically diagnosed HCMV retinitis without microbiology or pathological confirmation of HCMV retinal or ocular infection in AIDS patients [39, 40, 41]. The latter exclusion is due to the possibility that some AIDS patients may have had other infections, such as toxoplasmosis [42] or herpetic retinopathy [43], which can mimic HCMV infection involving ON.

### 6.1 Prevalence of Postnatal HCMV‐Associated Optic Neuropathies

HCMV infection rarely involves the ON, although it frequently infects the eyes particularly in AIDS or other immunocompromised patients. The prevalence of HCMV ocular involvement in AIDS patients was as high as 46%, including 20%–30% of HCMV retinitis, prior to the advent of antiretroviral therapy. However, with the appropriate use of antiretroviral treatment, this prevalence has significantly decreased to < 10% in recent years [1, 11, 39, 41]. HCMV ocular disease occurs in ~1%–6% of other immunocompromised patients with HCMV viremia or cHCMV cases; it is rare in immunocompetent individuals and HCMV optic neuropathy is extremely rare [38]. When HCMV ocular infection occurs, it can manifest as corneal endotheliitis, keratouveitis, retinitis, retinal branch angiitis or anterior/distal optic neuropathy (papillitis) [1, 11]. While HCMV retinitis is the most common ocular infection with a substantial risk of vision loss, papillitis coexisting or associated with retinitis occurs in 4%–14% of immunocompromised patients and is linked to a poorer prognosis [39, 41]. A major challenge in analyzing the prevalence or incidence of HCMV optic neuropathies is the lack of microbiological or pathological confirmation of HCMV infection in some earlier studies. Several studies from the 1980s to the 1990s, involving 4 to 22 AIDS patients with papillitis each, based the diagnosis of HCMV papillitis solely on clinical and funduscopic findings, without detecting HCMV in tissue or body fluids [39, 40, 41]. This diagnostic approach in those studies may have led to an overestimation of the prevalence of HCMV‐associated optic neuropathies, as some optic neuropathies associated with non‐ocular HCMV infection could be immune‐mediated or ischemic/secondary rather than due to direct infection (Table S1; as discussed later in Pathogenesis).

### 6.2 Pathogenesis of Postnatal HCMV‐Associated Optic Neuropathies

The pathogenic mechanisms underlying optic neuropathies are diverse and may be infectious or non‐infectious [1, 44, 45, 46, 47, 48, 49, 50, 51]. Given the aforementioned protective mechanisms of the PNS, HCMV infection of the ON is rarely seen in immunocompromised patients and exclusively in continuity with HCMV infection of the adjacent tissue particularly the eye or brain [52, 53]. An autopsy study of 54 AIDS patients with HCMV infection found no cases of HCMV optic neuritis without ocular involvement [53]. Of these patients, 20% had HCMV retinitis, 9% had both ocular and cerebral infections, 13% had HCMV infection limited to the eyes, and no cases showed HCMV infection confined to the brain. This study suggests that retrograde viral spread from the brain through ON appears to be an infrequent mechanism of HCMV retinal or ocular infection [40, 53]; however, this mechanism has not been confirmed by pathological case examinations [54, 55, 56]. In contrast, HCMV antegrade spread from the retinal or ocular infection to the ON head is a pathologically confirmed mechanism, demonstrated in a few post‐mortem cases examining the ON, ocular and brain tissues [52, 57]. Nevertheless, in many reported cases, HCMV papillitis was diagnosed by funduscopic and clinical features, with HCMV detected in the CSF, blood or ocular tissue, but without microbiological or pathological confirmation of HCMV presence within the ON (Table S1). These cases include several reports of HCMV‐associated papillitis in immunocompetent patients [46, 47, 48, 49]. A recent case report described an immunocompetent patient who initially had HCMV retinitis in one eye, and 7 weeks later developed HCMV optic neuritis in the contralateral eye, which was attributed to viral transmission via the CSF [46]. In these cases, there are pathogenic concerns about optic neuropathies which pathological changes (identified on funduscopic or pathological examination in some cases) may be secondary or reactive to HCMV retinitis and/or non‐infectious immune‐mediated (Table S1). At least one case of ischemic optic neuropathy has been identified through radiodiagnostic imaging and confirmed by post‐mortem examination, revealing the ON infarction secondary to systemic HCMV infection, with HCMV found in the brain but not in ON [54].

While the non‐infectious mechanisms underlying optic neuropathies are diverse, those following or concurrent with HCMV systemic infection, such as optic neuritis without direct ON infection, are typically immune‐mediated neuropathies and are often associated with an autoimmune disease [44, 45]. This pathogenic mechanism has been demonstrated in at least three cases [50, 51, 55], and it cannot be excluded in many other cases without evidence of the ON HCMV infection, as discussed above [44, 45]. In some autoimmune optic neuropathies, including HCMV‐associated cases [51, 58], there are targetable glial and neuronal antigens such as AQP4 and MOG. Autoantibodies against these extracellular plasma membrane proteins, AQP4 and MOG, are capable of mediating tissue injury through multiple effector functions including complement‐dependent cytotoxicity, antibody‐dependent cell‐mediated cytotoxicity, and antibody‐dependent cell‐mediated phagocytosis [44]. AQP4‐IgG in NMOSD can trigger multiple pathological responses, resulting in glial degeneration and tissue injury in the ON with secondary demyelination [44, 59]. Similarly, MOGAD shows evidence of both antibody‐dependent and antibody‐independent immunopathology, including complement deposition, inflammatory CD4‐predominant T cell infiltrates, granulocytosis, astrogliosis, microglial activation, and moderate axonal loss, along with the CNS perivenous and confluent demyelination [44, 60]. Despite these observations, the immune mechanisms of HCMV‐associated optic neuropathies may be more complex and remain elusive.

### 6.3 Clinical Manifestations of Postnatal HCMV‐Associated Optic Neuropathies

Optic neuropathies typically manifest with decreased visual acuity, altered colour vision, and abnormal visual fields in the affected eye, regardless of the infectious or non‐infectious etiopathogenetic mechanism [45]. The previously reported cases (*n* = 32, in Table S1) of HCMV‐positive optic neuropathies were mostly seen in male patients (*n* = 22; 69%) and in immunocompromised patients (at least 63%; 16 ADIS and 4 other immunocompromised conditions). Nearly all of these immunocompromised cases (except one case listed in Table S1 [61]) presented as papillitis associated with HCMV‐induced juxtapapillary retinitis. This type of HCMV juxtapapillary retinitis/papillitis is a progressive disease, typically characterized by an early afferent pupillary defect and initially preserved visual acuity, followed by rapid deterioration despite prompt antiviral therapy [41]. A cohort study of HCMV papillitis in 22 AIDS patients (5 female and 17 male) showed that 11 patients presented with HCMV papillitis at initial diagnosis, while the other 11 patients developed papillitis following antiviral treatment of pre‐existing HCMV retinitis [39].

Approximately one third of reported HCMV‐related optic neuropathies have occurred in immunocompetent individuals, with most cases documented over the past 20 years (Table S1). In a few of these cases, previously immunocompetent patients received a course of immunosuppressant/immunomodulatory therapy for a presumed autoimmune etiology, after which they developed HCMV papillitis [48, 62, 63]. Despite the etiopathogenesis, clinical manifestations almost universally include a decrease to loss of vision in almost all cases and some other case‐based symptoms such as weakness in two NMO cases [50, 51]. HCMV papillitis with accompanying retinitis was occasionally misdiagnosed as an ischemic or another non‐infectious disease before HCMV was detected in patients [64]. In fact, true ischemic optic neuropathy occurring adjacent to HCMV infection has been rarely observed [54]. In a few cases, magnetic resonance imaging (MRI) demonstrated the optic neuropathy as a longitudinal or diffuse contrast‐enhancing lesion along the ON, with or without associated CNS and/or meningeal foci [43, 46, 50, 54, 55]. Nevertheless, in several other cases, the brain/orbital MRI was unremarkable (Table S1) [11, 47, 65].

### 6.4 Pathology of Postnatal HCMV‐Associated Optic Neuropathies

The diagnosis of optic neuropathies is readily made by pathologic examination, while it may also be suggested by radiodiagnostic imaging and/or funduscopic examination. On funduscopic examination, papillitis typically represents as a pale or swollen appearance of the distal ON head often accompanied by adjacent retinal changes such as necrosis and/or hemorrhage [39, 41, 43]. However, other segments particularly the proximal portion of ON are assessable only through radiodiagnostic imaging or pathologic examination usually performed on autopsy specimens. A few autopsy studies have examined the ON and adjacent structures in HCMV cases [41, 52, 54, 55, 56, 57]. Pathologic features of HCMV optic neuropathy include necrosis or microinfarction, degeneration with marked loss of nerve fibers, inflammatory cell infiltrates including T cells and macrophages, as well as reactive changes like gliosis or fibrosis (Figure 1F–K). An immunohistochemical study of 6 HCMV‐positive optic nerves in AIDS patients revealed the expression of TNF‐α in astrocytes with reactive changes, endothelial cells and microglia/macrophages, but not in oligodendrocytes [66], which suggested HCMV‐associated inflammatory and reactive or degenerative changes without primary demyelination. As aforementioned in pathogenesis, there have been extremely rare cases in which HCMV‐positive cells were identified in the ON head, likely due to an antegrade spread from the adjacent retinal HCMV infection [52, 57]. Pathologic changes in the other segment particularly the proximal portion of the ON may be degenerative and secondary or reactive to the retinal or brain pathology [54] and/or immune‐mediated often associated with CNS pathology as seen in MOGAD or NMOSD [44, 50, 51, 58]. The pathology of HCMV‐associated, immune‐mediated optic neuropathies remains poorly understood and requires further study.

### 6.5 Treatment and Outcomes

As most of the reported cases had other concomitant pathologies particularly in immunocompromised patients (Table S1), the treatment in those cases of HCMV‐associated optic neuropathies was complicated by additional targets including HIV and other pathogens. The treatment focusing on HCMV typically involves intravenous antiviral agents such as ganciclovir and/or foscarnet. Visual outcomes vary widely. Approximately one‐third of reported cases experienced partial recovery, and about 20% achieved full visual recovery. However, fatal outcomes or persistent visual deficits were common, particularly in cases involving retrobulbar or ischemic optic neuropathy [43, 54, 65]. In a cohort of AIDS patients with HCMV papillitis, aggressive intravenous antiviral therapy led to resolution in the majority of patients [39]. However, in other cases, particularly those complicated by systemic disease or immunosuppressive therapy, visual outcomes were poor. The use of immunomodulatory therapy in suspected immune‐mediated optic neuropathies poses a clinical dilemma due to the risk of exacerbating latent HCMV infection. Treatment decisions in such cases must be individualized and carefully balanced.

## 7 HCMV‐Associated Vestibulocochlear Neuropathies and Other Cranial Neuropathies

Although cHCMV infection‐induced vestibulocochlear neuropathies are common, with a late‐onset SNHL occurring in a small percentage of cases during early childhood, postnatally acquired HCMV infection of the vestibulocochlear nerve has not been described. However, one case report described an auditory neuropathy in a very preterm infant with an HCMV‐positive urine culture [67]. A recent study examining serological infection parameters in 98 patients with vestibular neuropathy found only one case of HCMV IgM positivity, while 24 (45.3%) patients had elevated HCMV IgG levels [68]. It seems that HCMV‐associated vestibulocochlear neuropathies are more likely driven by inflammatory and immunological mechanisms, for which a causal treatment may be complemented [68].

There are only a few case reports of non‐optic cranial neuropathies (Table S2). A case report described palsy of multiple cranial nerves, including VI, IX, X and XII, in an HIV‐positive patient with HCMV‐positive serology and brain lesions [69]. A case series of 7 patients with isolated cranial neuropathy found 2 cases with associated HCMV infection evidenced by positive HCMV IgM in the serum. One case showed bilateral trigeminal nerve neuropathy (involving all three branches), while the other presented with bilateral facial nerve neuropathy; additionally, both cases tested positive for anti‐glycolipid antibodies in the serum, suggesting an immune‐mediated neuropathy [70]. In addition, there are a few reported cases of GBS or multifocal neuropathy with associated facial nerve palsy [13, 71, 72]. In these cases, the dominant pathogenic mechanism is presumably immune‐mediated, likely in association with HCMV infection, for which antiviral and immunomodulating agents are empirically administered.

## 8 HCMV‐Associated Spinal Neuropathies

HCMV‐associated spinal neuropathies are primarily immune‐mediated, as only a few cases of AIDS patients have shown HCMV antigens in the spinal nerve roots and the majority of spinal neuropathies have had no HCMV detected in the nerve tissue despite the presence of systemic HCMV infection [4, 73]. Immune‐mediated neuropathies consist of a heterogeneous spectrum of peripheral nerve disorders [73, 74, 75]. Pathologically, these neuropathies are characterized by immune or inflammatory cell infiltrates, myelin loss and/or axonal degeneration.

The most well‐studied HCMV‐associated immune‐mediated neuropathy is GBS (Table S2). Concurrent or preceding HCMV infection is observed in 4%–15% of GBS/AIDP patients [76, 77, 78], with higher percentages in patients with hematopoietic stem cell transplantation [73] or organ transplantation [79], and in patients with anti‐GalNAc‐GD1a antibodies [71], as well as in pregnant GBS patients [78]. An international study of 768 GBS patients with preceding infections, including 30 HCMV‐positive cases, found that sensory symptoms were most common, where the sensorimotor variant accounted for 94% of cases, while the remaining cases were pure motor variants [76]. Electrophysiologically, 86% of cases were classified as demyelinating, 7% as axonal, and the rest as equivocal [76]. Concurrent or preceding HCMV infection with CIDP is rarely reported in patients with hematopoietic stem cell or organ transplantation (Table S2). These HCMV‐positive CIDP patients exhibited more motor than sensory manifestations on neurologic examination, with electromyographic evidence of denervation [80]. Given the pathogenic interplay between HCMV infection and the immune‐mediated process, the treatment of these spinal neuropathies may involve a combination of antiviral and immunomodulating agents, often alongside other adjuvant therapy. It has been noted that HCMV‐associated GBS patients seem to be younger and generally have a relatively better prognosis [79].

Acute lumbosacral polyradiculopathy in AIDS patients is considered an uncommon, distinctive neurologic syndrome and mostly HCMV‐associated with HCMV typically detected in the CSF [12, 14, 81]. Its clinical presentation is characterized by rapidly progressive bilateral leg weakness, potentially leading to flaccid paraparesis, often accompanied by pain and paresthesia of the legs and perineum, areflexia, and sphincter dysfunction. MRI of the cauda equina and lumbosacral roots and/or myelography may appear normal, but electromyography of the affected muscle can reveal acute denervation that is also evident on post‐mortem examination in some patients [12] and predominantly a motor axonal neuropathy [81]. While this type of HCMV‐associated acute polyradiculopathy typically involves lumbosacral nerves, cervical nerves may also be affected, as described in at least two reported cases [81, 82]. Acute cervical polyradiculopathy may be pathogenically different, with a more immune‐mediated process, and clinically manifests as bilateral pain and weakness in the arm muscles [82]. The empirical treatment of HCMV‐associated acute polyradiculopathy involves antiviral therapy with adjuvant treatments. The prognosis of acute lumbosacral polyradiculopathy in AIDS patients is generally poor, with an overall median survival of 4 months. Death often occurs due to other opportunistic infections rather than polyradiculopathy itself [12]. However, the prognosis is better, with full recovery, in an immunocompetent patient with acute cervical polyradiculopathy [82].

## 9 HCMV‐Associated Peripheral/Distal Neuropathies

The reported peripheral/distal neuropathies associated with extraneurologic HCMV infection are mostly unclassified (or neuropathies, not otherwise specified, NOS), with various pathogenic mechanisms (Table S2). While they are often associated with encephalitis or myeloradiculitis [83, 84, 85], there have been multiple cases of isolated peripheral neuropathies in association with extraneurologic HCMV infection (Table S2). A recent study of cancer‐related peripheral neuropathies in patients with ovarian cancers treated with chemotherapy found that 38.8% (59/152) of patients with peripheral neuropathies were plasma HCMV‐positive. These patients experienced significantly more symptoms of peripheral neuropathies compared with HCMV‐negative patients [86]. Another study of 47 adult immunocompetent patients with HCMV hepatitis revealed a peripheral neuropathy in 4.3% (2/47) of patients [87]. HCMV‐associated peripheral neuropathies may present as polyneuropathy or multiple mononeuropathies, particularly when HCMV is detected in the CSF and/or blood [88, 89]. These neuropathies can be painful or painless, with neurological symptoms including sensory loss and/or distal weakness; they can be sensorimotor neuropathies or purely sensory neuropathies [90]. Nerve conduction study may reveal reduced sensory nerve action potential [89, 90] or multifocal demyelination and axonal loss in the affected nerves [88].

Pathological studies have been occasionally conducted on the affected nerves. An autopsy study of 4 out of 5 AIDS patients identified HCMV‐positive cells in the lower spinal cord as well as in the endoneurium and its capillary of the peroneal nerves. These nerves exhibited multiple foci of endoneurial necrosis and inflammatory infiltrates including mononuclear and neutrophilic polymorphonuclear cells, along with mixed axonal and demyelinating lesions of nerve fibers [5]. However, in some other cases, HCMV‐infected cells were found only within the endothelial cells of small blood vessels in the epineurium surrounding the peripheral nerve and dorsal root ganglia, but not in the nerve fibers or endoneurium/perineurium [89, 91, 92]. In a few case reports of HCMV‐associated neuropathies, including one with multiple mononeuropathies, the sural nerves exhibited axonal degeneration with loss of nerve fibers [89, 91], and occasionally mixed demyelination, with or without vasculitic changes [92, 93]. The pathogenic mechanisms in peripheral neuropathies include direct HCMV infection [5], as well as non‐infectious processes such as vasculitis, toxicity, autoimmune processes, and degenerative changes including Wallerian degeneration (Table 1) [86, 93, 94, 95, 96]. In the cases described, HCMV‐associated peripheral neuropathies were routinely treated with antiviral agents, with or without steroids, immunoglobulin, and/or adjuvant therapy; their outcomes were variable, ranging from improvement in cases of isolated peripheral neuropathies [89, 93, 94] to death in cases involving HCMV infection of the CNS and other organs [19, 91].

## 10 Conclusion

HCMV‐associated neuropathies represent a pathogenically heterogeneous group of PNS disorders, which include direct HCMV infection of the involved nerve, as well as non‐infectious pathologies such as axonal or degenerative, vasculitic/ischemic or necrotizing, inflammatory demyelinating, and/or immune‐mediated neuropathies (Figure 1). The immune‐mediated mechanism is predominant in certain types of HCMV‐associated neuropathies such as inflammatory demyelinating neuropathies and other antibody‐associated neuropathies. It may also play a variable role in other HCMV‐associated neuropathies such as vasculitic neuropathy. cHCMV‐associated neuropathies are primarily cochlear/auditory and optic neuropathies, with somewhat different pathogenesis compared with their postnatal counterparts, given the difference between the fetal and adult immune systems in response to HCMV infection. HCMV‐associated optic neuropathies warrant more attention, as they are frequently linked to pathologies of the retina and/or CNS with HCMV predilection. Clinical manifestations of these neuropathies are variable, but often present with weakness, pain or sensory deficits with electrophysiological and/or pathological evidence of axonal degeneration and/or demyelination. Given the multiple pathogenic mechanisms with variable autoimmunity in HCMV‐associated neuropathies, empirical treatment in these neuropathies typically involves antiviral therapy often combined with immunomodulatory treatment on a case‐by‐case basis. Prompt and appropriate treatment of these HCMV‐associated neuropathies can improve patient outcomes.

## Conflicts of Interest

The authors declare no conflicts of interest.

## Supporting information


**Table S1:** Reported cases of cytomegalovirus‐associated optic neuropathy.

**Table S2:** Cytomegalovirus infection‐associated spinal and peripheral neuropathies (excluding optic neuropathies listed in Table 1).

## Data Availability

The data that support the findings of this study are available from the corresponding author upon reasonable request.
