# Supplementary material for: Human Cytomegalovirus Associated Neuropathies: A Comprehensive Review From Pathophysiology to Clinical and Therapeutic Considerations
Source: J Peripher Nerv Syst. 2025 Dec 8;30(4):e70087. doi: 10.1111/jns.70087 (PMC12686851; doi:10.1111/jns.70087)
Supplement: Supplementary file 1 — Table S1: Reported cases of cytomegalovirus‐associated optic neuropathy. [file JNS-30-0-s002.docx]

**Table 1. Reported cases of cytomegalovirus-associated optic neuropathy**

| **Case [Ref]** | **Age (yrs), M/F** | **Clinical presentation** | **PMH/ /comorbidity** | **Imaging** | **Diagnosis** | **CMV detection** | **CMV involved organ(s)** | **Pathologic features** | **Treatment** | **Outcome** |
| --- | --- | --- | --- | --- | --- | --- | --- | --- | --- | --- |
| Present case | 28+3 gestational wks, M | Premature birth requiring resuscitation; death on day 1 of life | Antenatal non-immune hydrops including scalp edema, echogenic bowel, pericardial effusion, ascites with hepatomegaly & anhydramnios | N/A | Optic neuropathy a-w systemic CMV infection | CMV inclusion & IHC+ in multiple organs including kidney & brain (Figure 1) | Lung, liver, thyroid, kidney & brain | Autopsy ON, proximal: marked loss of nerve fibers; inflammatory cell infiltrates (CD68+ macrophages & CD3+ or CD8+ T-cells; gliotic/reactive changes | N/A | Died |
| Takahashi, et al. 2024 [1] | 63, M | Blurred vision in R.E.; 7 wks later, blurred vision in L.E. | Cataract surgery in R.E. 1 yr ago; intrascleral fixation of an intraocular lens, 8 mo ago; & vitrectomy for retinal detachment 2 mo ago | MRI, brain/orbit: unremarkable; 7 wks later, a longitudinal enhancing lesion of the left orbital nerve, suggesting optic neuritis | Left optic neuritis, following right CMV retinitis | Aqueous humor: R.E. CMV+ (by PCR; 6.06x10^4^ copies/mL); 7 wks later, R.E. CMV+ (1.30x10^4^ copies/mL) & L.E.-; CSF CMV+ | R.E. (with negative L.E.), CSF (with negative blood) | N/A | IV ganciclovir x3 wks & 2 courses of 3-day high-dose corticosteroid | Partial recovery of visual acuity & field |
| Alcibahy, et al. 2024 [2] | 41, F | Acute onset blurred vision in L.E. x25 days | Mild flu-like illness one 1 wk ago; left-sided headache | MRI: post-contrast diffuse enhancement of left ON; normal brain | Papillitis, a-w non-necrotizing viral retinopathy | Serum: CMV IgM & IgG highly+ | Circulatory system | N/A | Valganciclovir x 4 wks | Visual acuity improved &  maintained at 20/25 one mo later |
| Kaluarachchi & Abeykoon. 2022  [3] | 62, F | L.E. blurry vision with floaters, mild redness & pain | DM; severe COVID pneumonia treated with steroids & antibiotics x2 wks | MRI brain & orbit: normal | Optic neuropathy with Candida & CMV infection | Vitreous fluid: CMV+ by PCR); also Candida+ (by culture & smear) | Eye | N/A | Intravitreal amphotericin & ganciclovir; IV liposomal amphotericin B; oral voriconazole & oral valganciclovir | Vision improved in 1-3 wks |
| Melancia, et al. 2021  [4] | 24, F | Right ocular pain, visual loss with altered colour perception & paracentral scotoma x3 days; low-grade fever, malaise, fatigue & a painful cervical node x3 wks | Previously healthy | MRI, brain & orbit: no identifiable lesions | Optic neuropathy (without  evidence of retinitis, vasculitis or choroiditis) | CSF CMV+ (by PCR); serology IgG+; IgM- | CSF, circulatory system | N/A | IV Ganciclovir x14 days; IV corticosteroids (1 g methylprednisolone) x3 days | Visual function improved atter 2 wks; stable 8 wks after beginning of symptoms |
| O’Brien, et al. 2021  [5] | 43, M | Painless blurred vision & flashing lights in R.E. x10 days, with flu-like illness & fever | Pneumonia 6 mo ago; a corrective surgery for strabismus in R.E. in childhood | MRI, brain & orbit: normal | Papillitis | Serology: CMV IgM+, IgG+; PCR+ (2,334 copies/mL); CSF- | Circulatory system | N/A | Valganciclovir x3 wks | Recovery at 4 wk F-U ; 1 yr F-U: minimal residual visual deficit; clinically well |
| Du, et al. 2022  [6] | 39. M | Blurred vision in L.E. x1 mo; progressive, & blurring vision in R.E. | AIDS, EBV infection | N/A | Optic neuropathy, left, a-w necrotizing retinitis | Aqueous CMV viral load+ (4557 copies/ml in R.E.) | Eye | N/A | IV & binocular intravitreal ganciclovir | N/A |
| Du, et al. 2022  [6] | 27, M | Blurred vision, central vision loss & scotoma in both eyes x3 wks | AIDS, pulmonary infection, oral fungal infection | N/A | Optic neuropathy, left, a-w necrotizing retinitis | Aqueous CMV viral load+ (3859 copies/ml in R.E.; 4572 copies/ml in R.E. in L.E.) | Eye | N/A |  | N/A |
| Du, et al. 2022  [6] | 37, M | Blurred vision in both eyes x1 mo; worsen x1 wk | AIDS, oral fungal infection | N/A | Optic neuropathy, left, a-w necrotizing retinitis | Aqueous CMV viral load+ (13,640 copies/ml in L.E.) | Eye | N/A | IV intravenous ganciclovir & foscarnet; L.E. intravitreal ganciclovir injection | N/A |
| Luo, et al. 2020  [7] | 40, F | Intractable  nausea and dizziness x 9 wks, followed by dysphagia, limb weakness; R.E. blurred  vision & ghosting | Previously healthy | Brain MRI: T2-weighted hyperintense lesions on the medulla oblongata, corpora quadrigemina & right fourth ventricle wall (shrunk after antiviral treatment) | Cytomegalovirus (CMV)+, AQP4-IgG+  NMO | CSF: CMV+ (by pathogen gene sequencing); rectal biopsy: CMV+ (by IHC) | CSF, rectal tissue | N/A | Ganciclovir x wks | Recovery of R.E. visual acuity & neurologic improvement at 4 wks (with CSF: CMV- & AQP4-) |
| Rusescu, et al. 2017  [8] | 39, M | R.E. subacute pain & blurred vision | Anti-myelin oligodendrocyte glycoprotein (anti-MOG) antibody+ | MRI, brain/eye: longitudinally extensive involvement of the intraorbital  segment of the right ON, & one periventricular white matter lesion; spinal cord: normal | Optic neuritis with papillitis; non-specific brain MRI findings (absent/- CSF oligoclonal bands; absent/-AQP4 antibodies) | Serum: anti-CMV IgM+, with low  avidity IgG & low CMV viral load | Circulatory system | N/A | Ganciclovir IV x4 days; Valganciclovir x 2 wks; Methylprednisolone IV x3 days | Full recovery of visual acuity; MRI at 6 mo: improvement in right optic neuritis |
| Chang et al. 2012  [9] | 33, F | Pain in both eyes, a-w headache & tinnitus x3 mo | Previously immunocompetent | N/A | Papillitis, bilateral, a-w panuveitis | Serum CMV+ | Circulatory system | N/A | Valganciclovir x14 days | Full recovery after 14-day antiviral therapy; 15 mo F-U: no systemic or ocular symptoms |
| Zheng, et al. 2012  [10] | 33, M | Abrupt decrease in bilateral vision | Lymphoblastic leukemia with CNS & bone marrow relapse, treated with hematopoietic stem cell  Transplantation & chemotherapy; CMV viremia | Brain MRI: unremarkable | Retrobulbar  optic neuritis | CSF & blood CMV+ (by | CSF & circulatory system | N/A | Antiviral  therapy (foscarnet 90 mg/kg, twice daily) & methylprednisolone  pulse therapy (500 mg/day for 3 days) | No improvement; almost total bilateral blindness |
| Park, et al. 2008  [11] | 34, M | Increasing shortness of breath a-w non-productive cough & fever x 2 wks; 5 days later, R.E. decreased vision (20/200); ophthalmoscopy: R.E. multiple yellow–white infiltrations & granular lesions; L.E. disc hemorrhage | AIDS | MRI, brain/ON: meningeal enhancement & increased  signal intensity along the optic tract & geniculate body | Retrobulbar optic neuritis, bilaterally, a-w retinal vasculitis & meningoencephalitis | CSF CMV+ (by PCR) wks after vitrectomy | CSF | N/A | Ganciclovir x wks | Neurologically/ ophthalmologically stable > 6 mo |
| Ioannidis, et al. 2008  [12] | 49. M | R.E. visual loss in x 3 days, with an ipsilateral swollen optic disc | AIDS | Brain MRI: normal | Juxtapapillary CMV retinitis, with optic neuritis | CSF: CMV+ (by PCR): | CSF | N/A | Valganciclovir > 2mo | Visual improvement, with complete resolution of peripapillary retinitis, 4-8 wks later |
| De Silva, et al. 2008  [13] | 22, F | Fever, headache, & blurred vision x3 wks; visual acuities: 20/60 in R.E. &  20/30 in L.E.; ophthalmoscopy: bilateral optic disc swelling with no evidence of retinitis | N/A; unremarkable neurologic examination | Brain MRI: normal; CT of chest, abdomen, and pelvis: normal | Papillitis (without  evidence of retinitis) | Serum:+ CMV IgM; IgG; PCR+ (CSF- for CMV & other pathogens) | Circulatory system | N/A | IV ganciclovir x2 wks | , normal visual acuities & color vision 2 mos later |
| Furukawa, et al. 2007  [14] | 54, F | Decreased vision in L.E. 3 months after IV triamcinolone  for the treatment of recurred macular edema | Well-controlled type 2 DM | N/A | Papillitis, a-w anterior  uveitis & retinal vasculitis | Vitreous: CMV+ (by PCR) | Eye (no systemic CMV) | N/A | IV ganciclovir (500mg) x 2 wks & intravitreous foscarnet, followed by vitreous surgery | Visual acuity improving 2 mo later |
| Tran, et al. 2007  [15] | 34, M to F (transsexual) | Diarrhoea, myalgia and weakness x8 wks; painless sudden loss of vision in R.E. | Immunocompetent  transsexual status; rhabdomyolysis | MRI, ON: increased T2 signal intensity with contrast enhancement in its intraorbital segment; brain: normal; spinal cord: T2-6 iincreased T2-signal  intensity | NMO following CMV primo-infection | Serum: CMV specific IgM+ & IgG+, with PCR for CMV DNA- (suggestive of a recent CMV  Infection) | Circulatory system (recent infection) | N/A | Valgancyclovir (900 mg b.i.d.) x 3 wks, mycophenolate mofetil, &  methylprednisolone x wks | Neurological symptoms improved in wks |
| Cackett, et al. 2004  [16] | 25, F | R.E. blurry vision, binocular diplopia, & bilateral supraorbital & frontal headache x1 mo;  bilateral lower limb weakness x2 wks ago | AIDS, newly diagnosed, with esophageal candidiasis | N/A | CMV-associated right optic neuropathy without retinitis, with right oculomotor nerve palsy & a lower limb polyradiculomyelopathy | CSF & blood: CMV+ (by PCR) | CSF, circulatory system | N/A | Zidovudine, lamivudine, abacavir & IV ganciclovir followed by twice monthly infusions of cidofovir with G-CSF; IV methylprednisolone x4 days & x3 days | Visual acuity & field improvement at 2 mo; with almost complete resolution of right oculomotor nerve palsy & lower limb weakness |
| Baglivo, et al. 1996  [17] | 32, F | L.E. progressive loss of vision & frontal headache x1 mo; several days later, R.E. vision worsened | N/A | N/A | Serology: anti-CMV IgM+ & IgG+; urine: early CMV antigen+ | Serology CMV+ | Circulatory system | N/A | Foscarnet x3 wks; IV methylprednisolone x5 days followed by oral prednisone | Visual acuity resolution after 3 wks treatment |
| Mansour 1990  [18] | 34, M | N/A | AIDS, CMV pneumonitis, pneumocystis pneumonia, oral candidiasis, HIV encephalopathy | N/A | Left CMV optic neuritis | Serum: CMV+ | Circulatory system, lung | N/A | N/A | Died at 10 mo after eye exam |
| Mansour 1990  [18] | 38, M | N/A | AIDS, brain & lung CMV infection, oral candidiasis | N/A | Left CMV optic neuritis | Serum: CMV+ | Circulatory system, lung, brain | N/A | N/A | Died at 4 mo after eye exam |
| Grossniklaus, et al. 1987  [19] | 33, M | Decreased vision (20/60) in L.E. Ophthalmoscopy: L.E. peripapillary retinitis with focal hemorrhage, necrosis, & pale/swollen ON; R.E. focal retinitis with vascular tortuosity | AIDS | N/A | Left optic neuritis, a-w bilateral retinitis | CMV inclusions (IHC+) in all layers of the involved retinas & ON | Eye, ON head | Post-mortem, ON: mononuclear inflammatory cell infiltrate & CMV inclusions with CMV IHC+ cells | N/A | Died |
| Palestine, et al. 1986  [20] | 42, M | R.E. decreased visual acuity, progressed over 2 weeks to no light perception, & to L.E. | AIDS, *pneumocystis* *carinii* pneumonia,  *Histoplasma* pneumonia, Kaposi's sarcoma,&  Candida esophagitis | N/A | Retinitis, involving ON | Blood & urine cultures: CMV+ | Circulatory system, urinary tract | N/A | Dihydroxypropoxymethyl guanine | Temporary improvement 3 wks later; relapse leading to no light perception in L.E.; died wks later |
| Robinson et al. 1986  [21] | 32, M | L.E. decreased vision | Homosexual status, *pneumocystis* *carinii pneumonia* & disseminated CMV disease (AIDS like) | N/A | Retinitis, involving the ON head (papillitis) | Blood & urine cultures: CMV+ | Circulatory system, urinary tract | N/A | Dihydroxy propoxymethyl guanine | Visual improvement after 7 days |
| Rosecan, et al. 1986  [22] | 23, M | Progressively decreasing vision in R.E.; 3 mo later, decreasing vision in L.E. | AIDS; CNS toxoplasmosis with seizures, 6 mo ago | N/A | Retinitis, involving the ON head (papillitis) | Urine culture & a colonic biopsy: CMV+ | Urinary tract (or circulatory system), colon | N/A | Dihydroxy propoxymethyl guanine | 4 mo later, ophthalmologically stable; died after discontinuity of treatment; 6 mo later |
| Rosecan, et al. 1986  [22] | 45, M | Blurred version in L.E.; 1 mo later, in R.E. | AIDS; CNS toxoplasmosis 3 mo ago | N/A | Retinitis, involving the ON head (papillitis) | Urine culture: CMV+ | Urinary tract (or circulatory system), colon | N/A | Dihydroxypropoxymethyl guanine | Persistent poor version with no light perception bilaterally; died 5 mo later |
| Pepose et al. 1984  [23] | 44, M | Visual field defect & decreased visual acuities in L.E. & later R.E. | AIDS with multiple infections including *Pneumocystis carinii* pneumonia, CMV pneumonitis, herpetic oral & rectal ulcers, mucocutaneous candidiasis, & Kaposi's sarcoma | CT, brain: undiagnostic | CMV papillitis & retinitis | Retina, ON, brain | Brain (subependymal tissue), retina, ON head | Post-mortem, ON: mild to moderate degeneration & gliosis; IHC CMV+ (herpes simplex+ in the brain & retina but not in ON) | Acyclovir | Died in mos |
| Friedman, et al. 1983  [24] | 33, M | Blurred vision in L.E., a-w leucopenia & fever | AIDS, inflammatory colitis (with herpes simplex virus isolated from the perirectal ulcerations) | N/A | Retinitis, involving the ON head (papillitis) | Vitreous & urine cultures: CMV+ | Eye, CNS, liver, gastrointestinal tract & urinary tract (or circulatory system) | Post-mortem, ON head: swollen & inflammatory cell infiltrates; ocular tissue: necrosis, hemorrhage, leucocytes, & CMV intracytoplasmic & intra nuclear inclusions | N/A | Died 3 mo after admission |
| Marmor et al. 1978  [25] | 51, M | Decreased vision in L.E. | Lymphoma x7 yrs, treated with cyclophos phamide & vincristine, & prednisone; multiple skin carcinomas, disseminated herpes zoster & bacterial conjunctivitis in the prior yr | CT, brain: dilation of the ventricles & generalized cerebral atrophy | CMV papillitis | Ocular tissue CMV+ | Lungs, retina/eye (without involving brain or ON) | Post-mortem ON: thickened & necrotic; retinal cells containing CMV characteristic inclusions | Doxorubicin hydrochloride, cytarabine, & 6-thioguanine | Died in 3 wks |

Abbreviations: +, positive/present; -, negative/absent; AIDS, acquired immunodeficiency syndrome; AQP4, aquaporin4 antibodies; Ara-A, Vidarabine (or Adenine arabinoside); a-w, associated with; CMV, cytomegalovirus; COVID, coronavirus disease; CSF, cerebrospinal fluid; DM, diabetes; F, female; FITC, fluoroscein isothiocyanate; F-U, follow-up; G-CSF, granulocyte-colony stimulating factor; HIV, human immunodeficiency virus; Ig, Immunoglobulin; IHC, immunohistochemistry; IV, intravenous; L.E., left eye; M, male; MRI, magnetic resonance image; N/A, not available; NMO, neuromyelitis optica; ON, optic nerve; PCR, polymerase chain reaction; PMH, past medical history; R.E., right eye; Ref, reference; US, ultrasound; wk, week

**References:**

1. Takahashi S, Hashida N, Maruyama K, et al. Cytomegalovirus-Induced Optic Neuritis Through Cerebrospinal Fluid Viral Transmission in an Immunocompetent Patient: A Case Report. J Neuroophthalmol. 2024;44:e216-e218. doi: 10.1097/WNO.0000000000001834.
2. Alcibahy Y, Panwar N, Malik A, Agarwal A. Cytomegalovirus-Associated Non- Necrotizing Retinopathy, Occlusive Retinal Vasculitis, and Neovascularization. Ocul Immunol Inflamm. 2024;32:529-533. doi: 10.1080/09273948.2024.2325054.
3. Kaluarachchi S, Abeykoon M. A case of endogenous candida endophthalmitis with incidental cytomegalovirus infection and optic neuropathy in a patient recovered from severe COVID-19. Indian J Ophthalmol. 2022;70:323-326. doi: 10.4103/ijo.IJO_2454_21.
4. Melancia D, Fernandes A, Manita M, Cordeiro IM. Cytomegalovirus optic neuropathy in a young immunocompetent patient. J Neurovirol. 2021;27:364-366. doi: 10.1007/s13365-021-00963-3.
5. O'Brien TN, O'Connor GM, Lefter S. Papillitis, a Rare Cytomegalovirus Manifestation in an Immunocompetent Host. J Neuroophthalmol. 2021;41:e34-e35. doi: 10.1097/WNO.0000000000000925.
6. Du KF, Huang XJ, Chen C, Kong WJ, Xie LY, Wei WB. Clinical characteristics in the misdiagnosis of cytomegalovirus retinitis: A retrospective analysis of eight patients. Indian J Ophthalmol. 2022;70:3596-3602. doi: 10.4103/ijo.IJO_1761_21.
7. Luo J, Shi X, Lin Y, et al. Cytomegalovirus Infection in an Adult Patient With Neuromyelitis Optica and Acute Hemorrhagic Rectal Ulcer: Case Report and Literature Review. Front Immunol. 2020;11:1634. doi: 10.3389/fimmu.2020.01634.
8. Rusescu BV, Diederich NJ, Tsobo C, Marignier R, Kerschen P. MOG antibody-associated optic neuritis in the setting of acute CMV infection. J Neurol Sci. 2017;382:44-46. doi: 10.1016/j.jns.2017.09.011.
9. Chang PY, Hamam R, Giuliari GP, Foster CS. Cytomegalovirus panuveitis associated with papillitis in an immunocompetent patient. Can J Ophthalmol. 2012;47:e12-3. doi: 10.1016/j.jcjo.2012.03.011.
10. Zheng X, Huang Y, Wang Z, Yan H, Pan S, Wang H. Presumed cytomegalovirus-associated retrobulbar optic neuritis in a patient after allogeneic stem cell transplantation. Transpl Infect Dis. 2012;14:177-9. doi: 10.1111/j.1399-3062.2011.00678.x.
11. Park KH, Bang JH, Park WB, et al. Retrobulbar optic neuritis and meningoencephalitis following progressive outer retinal necrosis due to CMV in a patient with AIDS. Infection. 2008;36:475-9. doi: 10.1007/s15010-007-6290-x.
12. Ioannidis AS, Bacon J, Frith P. Juxtapapillary cytomegalovirus retinitis with optic neuritis. J Neuroophthalmol. 2008;28:128-30. doi: 10.1097/WNO.0b013e3181782e52.
13. De Silva SR, Chohan G, Jones D, Hu M. Cytomegalovirus papillitis in an immunocompetent patient. J Neuroophthalmol. 2008;28:126-7. doi: 10.1097/WNO.0b013e3181782fed.
14. Furukawa M, Kumagai K, Ogino N, Okinami S, Uemura A, Larson E. Cytomegalovirus retinitis after intravitreous triamcinolone treatment of a vitrectomized eye in an immunocompetent patient. Retin Cases Brief Rep. 2007;1:205-7. doi: 10.1097/ICB.0b013e31804d1e3f.
15. Tran C, Du Pasquier RA, Cavassini M, et al. Neuromyelitis optica following CMV primo-infection. J Intern Med. 2007;261:500-3. doi: 10.1111/j.1365-2796.2007.01794.x.
16. Cackett P, Weir CR, McFadzean R, Seaton RA. Optic neuropathy without retinopathy in AIDS and cytomegalovirus infection. J Neuroophthalmol. 2004 Mar;24(1):94-5. doi: 10.1097/00041327-200403000-00030.
17. Baglivo E, Leuenberger PM, Krause KH. Presumed bilateral cytomegalovirus-induced optic neuropathy in an immunocompetent person. A case report. J Neuroophthalmol. 1996;16:14-7.
18. Mansour AM. Neuro-ophthalmic findings in acquired immunodeficiency syndrome. J Clin Neuroophthalmol. 1990;10:167-74.
19. Grossniklaus HE, Frank KE, Tomsak RL. Cytomegalovirus retinitis and optic neuritis in acquired immune deficiency syndrome. Report of a case. Ophthalmology. 1987;94:1601-4. doi: 10.1016/s0161-6420(87)33261-0.
20. Palestine AG, Stevens G Jr, Lane HC, et al. Treatment of cytomegalovirus retinitis with dihydroxy propoxymethyl guanine. Am J Ophthalmol. 1986;101:95-101. doi: 10.1016/0002-9394(86)90470-8.
21. Robinson MR, Streeten BW, Hampton GR, Siebold EC, Taylor-Findlay C. Treatment of cytomegalovirus optic neuritis with dihydroxy propoxymethyl guanine. Am J Ophthalmol. 1986;102:533-4. doi: 10.1016/0002-9394(86)90088-7.
22. Rosecan LR, Stahl-Bayliss CM, Kalman CM, Laskin OL. Antiviral therapy for cytomegalovirus retinitis in AIDS with dihydroxy propoxymethyl guanine. Am J Ophthalmol. 1986;101:405-18. doi: 10.1016/0002-9394(86)90638-0.
23. Pepose JS, Hilborne LH, Cancilla PA, Foos RY. Concurrent herpes simplex and cytomegalovirus retinitis and encephalitis in the acquired immune deficiency syndrome (AIDS). Ophthalmology. 1984;91:1669-77.
24. Friedman AH, Orellana J, Freeman WR, et al. Cytomegalovirus retinitis: a manifestation of the acquired immune deficiency syndrome (AIDS). Br J Ophthalmol. 1983;67:372-80. doi: 10.1136/bjo.67.6.372.
25. Marmor MF, Egbert PR, Egbert BM, Marmor JB. Optic nerve head involvement with cytomegalovirus in an adult with lymphoma. Arch Ophthalmol. 1978;96:1252-4. doi: 10.1001/archopht.1978.03910060078016.
