# Supplementary material for: Human Cytomegalovirus Associated Neuropathies: A Comprehensive Review From Pathophysiology to Clinical and Therapeutic Considerations
Source: J Peripher Nerv Syst. 2025 Dec 8;30(4):e70087. doi: 10.1111/jns.70087 (PMC12686851; doi:10.1111/jns.70087)
Supplement: Supplementary file 2 — Table S2: Cytomegalovirus infection‐associated spinal and peripheral neuropathies (excluding optic neuropathies listed in Table 1). [file JNS-30-0-s001.docx]

**Table 2. Cytomegalovirus infection-associated spinal and peripheral neuropathies (excluding optic neuropathies listed in Table 1)**

| **Study [Ref]** | **CMV case(s)** | **Neuropathy/diagnosis (main)** | **Comorbidity** |
| --- | --- | --- | --- |
| Li et al. 2024 [1] | 59 (out of 152) | Peripheral neuropathy, NOS | Ovarian cancer |
| Toro et al. 2024 [2] | 1 | GBS | Status post liver transplantation |
| Krief et al. 2023 [3] | 5 (out of 16); 2 (out of 18) | GBS in pregnancy (5) versus non-pregnancy (2) | Pregnancy, with 2 fetuses died in 2/5 cases |
| Ginanneschi 2022 [4] | 5 (out of 84) | GBS | N/A |
| Scanvion & Morell-Dubois 2022 [5] | 1 | Neuralgic Amyotrophy | N/A |
| Komura et al. 2020 [6] | 2 (out of 47) | Peripheral neuropathy, NOS | Cytomegalovirus Hepatitis |
| Ren et al. 2019 [7] | 38 (out of 40) | Immune-mediated neuropathies: GBS (incl. Miller-Fisher syndrome) & unclassified peripheral neuropathies | Status post haploidentical hematopoietic  stem cell transplantation |
| Palma et al. 2018 [8] | 1 | Mononeuritis multiplex | AIDS with HIV encephalopathy & disseminated CMV disease (incl. esophagitis, colitis, encephalitis) |
| Alhefzi et al. 2016 [9] | 1 | GBS, axonal form | Status post facial allograft (CMV donor-seropositive, recipient-seronegative) transplantation |
| Shaban et al. 2016 [10] | 1 | GBS | Status post kidney transplantation |
| Vasanthan et al. 2015 [11] | 1 | Peripheral neuropathy, NOS | DRESS (drug reaction with eosinophilia & systemic symptoms); congenital CMV infection |
| Kameda et al. 2015 [12] | 3 | Polyradiculopathy | AIDS |
| Miyaji et al. 2014 [13] | 8 (out of 41) | AIDP (GBS) | N/A |
| Sawai et al. 2014 [14] | 6 (out of 40) | AIDP (GBS) | N/A |
| Press et al. 2014 [15] | 4 (out of 11) | CIDP | Autologous haematopoietic stem cell  transplantation; 1 of Epstein–Barr virus reactivation |
| Baerts & van Straaten 2010 [16] | 1 | Auditory neuropathy (retrocochlear) | Premature status |
| Joh et al. 2009 [17] | 1 | Peripheral neuropathy, Bortezomib-associated, with dorsal column degeneration | Multiple myeloma |
| Sohal et al. 2009 [18] | 1 | Lumbosacral polyradiculopathy | Congenital HIV infection |
| Furiya et al. 2008 [19] | 1 | GBS | Multiple IgM anti-ganglioside antibodies+ |
| Levy et al. 2005 [20] | 1 | Vasculitic peripheral neuropathy, mononeuritis multiplex | Mixed Cryoglobulinemia Type II |
| Kunishige et al. 2004 [21] | 1 | Isolated cranial neuropathy (V1,2,3, bilateral) | Anti-glycolipid antibody+ |
| Kunishige et al. 2004 [21] | 1 | Isolated cranial neuropathy (VII, bilateral) | Anti-glycolipid antibody+ |
| Mori et al. 2006 [22] | 1 | CIDP and Myasthenia Gravis | N/A |
| Karna et al. 2001 [23] | 1 | Multiple cranial neuropathy (VI, IX, X, XII) | HIV+ ,with CNS and leptomeningeal lesions |
| Calza et al. 2001 [24] | 1 | GBS with left facial nerve palsy | Chronic hepatitis C & advanced HIV infection |
| El-Sabrout et al. 2001 [25] | 5 | GBS | Solid organ transplantation |
| Hadden et al. 2001 [26] | 19 (out of 229) | GBS | N/A |
| Kaida et al. 2001 [27] | 14 (out of 29) | GMS with frequent facial & sensory deficits | anti-GalNAc-GD1a antibodies+ |
| Meyer et al. 2000 [28] | 1 | Polyneuropathy | Necrotizing vasculitis |
| Broccolo et al. 2000 [29] | 7 (out of 16) | Peripheral neuropathy with encephalitis | AIDS |
| Ogawara et al. 2000 [30] | 9 (out of 88) | Axonal GBS | Anti-ganglioside antibodies+ |
| Vanpee et al. 2000 [31] | 1 | Brachial plexus neuropathy | N/A |
| de Maar et al. 1999 [32] | 1 | CIDP | Status post kidney transplantation |
| Yuki et al. 1998 [33] | 23 (out of 26) | Chronic polyneuropathy | IgM anti-MAG/SGPG antibody+ |
| Harada et al. 1998 [34] | 1 | Mononeuritis multiplex with Horners syndrome | AIDS |
| Calore et al. 1998 [35] | 1 (out of 15) | Neuropathy with polyradiculitis & histopathological changes in the sural nerve biopsy | AIDS |
| Corral et al. 1997 [36] | 3 (out of 17) | Acute polyradiculopathies | HIV infection |
| Irie et al. 1996 [37] | 3 | GBS | N/A |
| Miller et al. 1996 [38] | 17 | CMV+ acute lumbosacral polyradiculopathy | AIDS |
| Visser et al. 1996 [39] | 20 (out of 134) | GBS | N/A |
| Ben-Smith et al. 1996 [40] | 2 (out of 6) | GBS with T-lymphocytes in the sural nerve biopsy | N/A |
| Fox et al. 1995 [41] | 12 (out of 111) | Polyradiculopathy, or peripheral neuropathy | AIDS |
| Kuller et al. 1995 [42] | 1 | GBS | Pregnancy |
| So & Olney 1994 [43] | 15 (out of 23) | CMV+ acute lumbosacral polyradiculopathy | AIDS |
| Roullet et al. 1994 [44] | 15 | Multifocal neuropathy | HIV infection |
| Mastroianni et al. 1994 [45] | 10 (out of 13) | Painful peripheral neuropathy | HIV infection |
| Morgello & Simpson. 1994 [46] | 1 | Multifocal demyelinating polyneuropathy | AIDS |
| Kim & Hollander. 1993 [47] | 2 | Polyradiculopathy | N/A |
| Fuller et al. 1993; 1989 [48] | 20 (out of 25); 8 (out of 13) | Painful peripheral neuropathy; non-painful peripheral neuropathy | AIDS |
| Fiala et al. 1993 [49] | 7 (out of 10) | Peripheral neuropathy | AIDS dementia; CMV retinitis |
| Gozlan et al. 1992 [50] | 5 (out of 14) | Peripheral neuropathy and/or myeloradiculitis with (3/5) or without (2/5) CMV encephalitis | AIDS |
| McCormick et al. 1992 [51] | 1 | Peripheral neuropathy, NOS | Rheumatoid arthritis, vasculitis, cutaneous ulceration, and digital gangrene |
| Beydoun. 1991 [52] | 1 | Polyradiculopathy, with spinal  cord and neuromuscular syndromes | AIDS with anal herpes simplex & Pneumocystis carznii pneumonia |
| Said et al. 1991 [53] | 4 | Multifocal neuropathy | AIDS |
| Grafe & Wiley. 1989 [54] | 4 (out of 27) | Nerve or root pathology with brain pathology (4/4) and spinal cord pathology (2/4) | CMV ventriculitis, meningitis, and/or spinal cord CMV infection |
| Robert et al. 1989 [55] | 1 | Peripheral neuropathy with CNS CMV infection & multifocal necrotizing retinopathy | AIDS |
| Winer et al. 1988 [56] | 11 (out of 99) | GBS | N/A |
| Snider et al. 1983 [57] | 3 (out of 18) | Peripheral neuropathy with disseminated CMV infection, encephalitis (3/3) & chorioretinitis (1/3) | AIDS |
| Crisp et al. 1983 [58] | 1 | Recurrent polyneuropathy | Infectious mononucleosis with Epstein-Barr virus+ |
| Doherty & Bradfield. 1981 [59] | 1 | Peripheral neuropathy | Polyarteritis nodosa |

Abbreviations: &, and; AIDS, acquired immunodeficiency syndrome; AIDP, acute inflammatory demyelinating polyneuropathy; CIDP, Chronic inflammatory demyelinating polyradiculoneuropathy; CNS, central nervous system GBS, Guillain-Barré syndrome (AIDP); HIV, human immunodeficiency virus; incl., including; N/A, not available; NOS, not otherwise specified; Ref, references [listed below]

**References:**

1. Li X, Argenta PA, Brown K, et al. Associations of cytomegalovirus infection with cancer-related cognitive impairment and peripheral neuropathy in ovarian cancer survivors. Gynecol Oncol. 2024;191:25-30. doi: 10.1016/j.ygyno.2024.09.016.
2. Toro J, Gaitán J, Medina T, Reyes S. Guillain-Barré syndrome following primary cytomegalovirus infection in a patient with liver transplantation. BMJ Case Rep. 2024;17(1):e255739. doi: 10.1136/bcr-2023-255739.
3. Krief N, Gabriel R, Cauquil C, et al. Clinical features and maternal and fetal outcomes in women with Guillain-Barré syndrome in pregnancy. J Neurol. 2023;270:4498-4506. doi: 10.1007/s00415-023-11808-w.
4. Ginanneschi F, Giannini F, Sicurelli F, et al. Clinical Features and Outcome of the Guillain-Barre Syndrome: A Single-Center 11-Year Experience. Front Neurol. 2022;13:856091. doi: 10.3389/fneur.2022.856091.
5. Scanvion Q, Morell-Dubois S. Neuralgic amyotrophy triggered by cytomegalovirus: to be aware of this clinical diagnosis. Neurocase. 2022;28:320-322. doi: 10.1080/13554794.2022.2085118.
6. Komura T, Kagaya T, Takayama H, et al. Clinical Features and Dynamics of T Cells-Related Markers in Immunocompetent Patients with Cytomegalovirus Hepatitis. Can J Gastroenterol Hepatol. 2020;2020:8874620. doi: 10.1155/2020/8874620.
7. Ren XY, Liu X, Huang QS, et al. Incidence, Risk Factors, and Outcome of Immune-Mediated Neuropathies (IMNs) following Haploidentical Hematopoietic Stem Cell Transplantation. Biol Blood Marrow Transplant. 2019;25:1629-1636. doi: 10.1016/j.bbmt.2019.04.021.
8. Palma P, Costa A, Duro R, Neves N, Abreu C, Sarmento A. Mononeuritis multiplex: an uncommon neurological manifestation of cytomegalovirus reactivation in an HIV-infected patient. BMC Infect Dis. 2018;18:554. doi: 10.1186/s12879-018-3501-2.
9. Alhefzi M, Aycart MA, Bueno EM, et al. Guillain-Barré syndrome associated with resistant cytomegalovirus infection after face transplantation. Transpl Infect Dis. 2016;18:288-92. doi: 10.1111/tid.12516.
10. Shaban E, Gohh R, Knoll BM. Late-onset cytomegalovirus infection complicated by Guillain-Barre syndrome in a kidney transplant recipient: case report and review of the literature. Infection. 2016;44:255-8. doi: 10.1007/s15010-015-0819-1.
11. Vasanthan T, Rajaguru G, Venkatesh C, Narayanan P, Gulati R, Toi PCh. DRESS Syndrome with Peripheral Neuropathy Due to Reactivation of Cytomegalovirus in a Child. J Glob Infect Dis. 2015;7:89-90. doi: 10.4103/0974-777X.157249.
12. Kameda K, Shirano M, Hadano Y, et al. Cytomegalovirus polyradiculopathy in three Japanese patients with AIDS. Intern Med. 2015;54:513-8. doi: 10.2169/internalmedicine.54.2438.
13. Miyaji K, Shahrizaila N, Umapathi T, Chan YC, Hirata K, Yuki N. Are ERM (ezrin/radixin/moesin) proteins targets for autoantibodies in demyelinating neuropathies? Hum Immunol. 2014;75:1089-91. doi: 10.1016/j.humimm.2014.09.010.
14. Sawai S, Satoh M, Mori M, et al. Moesin is a possible target molecule for cytomegalovirus-related Guillain-Barré syndrome. Neurology. 2014;83:113-7. doi: 10.1212/WNL.0000000000000566.
15. Press R, Askmark H, Svenningsson A, et al. Autologous haematopoietic stem cell transplantation: a viable treatment option for CIDP. J Neurol Neurosurg Psychiatry. 2014;85:618-24. doi: 10.1136/jnnp-2013-306014.
16. Baerts W, van Straaten HL. Auditory neuropathy associated with postnatally acquired cytomegalovirus infection in a very preterm infant. BMJ Case Rep. 2010;2010:bcr0120102689. doi: 10.1136/bcr.01.2010.2689.
17. Joh T, Sigematu K, Yasui JI, et al. Dorsal Column Degeneration after Bortezomib Therapy in a Patient with Multiple Myeloma. Case Rep Oncol. 2009;2:184-188. doi: 10.1159/000231996.
18. Sohal A, Riordan A, Mallewa M, Solomon T, Kneen R. Successful treatment of cytomegalovirus polyradiculopathy in a 9-year-old child with congenital human immunodeficiency virus infection. J Child Neurol. 2009;24:215-8. doi: 10.1177/0883073808322671.
19. Furiya Y, Hirano M, Kusunoki S, et al. Complete recovery of an aged patient with Guillain-Barré syndrome associated with multiple IgM anti-ganglioside antibodies. Muscle Nerve. 2008;38:1630-3. doi: 10.1002/mus.21131.
20. Levy Y, Uziel Y, Zandman G, et al. Response of vasculitic peripheral neuropathy to intravenous immunoglobulin. Ann N Y Acad Sci. 2005;1051:779-86. doi: 10.1196/annals.1361.121. Erratum in: Ann N Y Acad Sci. 2012;1270:120.
21. Kunishige M, Mitsui T, Yoshino H, et al. Isolated cranial neuropathy associated with anti-glycolipid antibodies. J Neurol Sci. 2004;225:51-5. doi: 10.1016/j.jns.2004.06.017.
22. Mori M, Kuwabara S, Nemoto Y, Tamura N, Hattori T. Concomitant chronic inflammatory demyelinating polyneuropathy and myasthenia gravis following cytomegalovirus infection. J Neurol Sci. 2006;240:103-6. doi: 10.1016/j.jns.2005.08.013.
23. Karna S, Biswas J, Kumarasamy N, Sharma P, Solomon S. Multiple cranial nerve palsy in an HIV-positive patient. Indian J Ophthalmol. 2001;49:118-20.
24. Calza L, Manfredi R, Marinacci G, et al. Role of gancyclovir and HAART administration in the treatment of a rare complication of HIV disease: cytomegalovirus-associated Guillain-Barré syndrome. J Chemother. 2001;13:575-7. doi: 10.1179/joc.2001.13.5.575.
25. El-Sabrout RA, Radovancevic B, Ankoma-Sey V, Van Buren CT. Guillain-Barré syndrome after solid organ transplantation. Transplantation. 2001;71:1311-6. doi: 10.1097/00007890-200105150-00023
26. Hadden RD, Karch H, Hartung HP, et al; Plasma Exchange/Sandoglobulin Guillain-Barré Syndrome Trial Group. Preceding infections, immune factors, and outcome in Guillain-Barré syndrome. Neurology. 2001;56:758-65. doi: 10.1212/wnl.56.6.758.
27. Kaida K, Kusunoki S, Kamakura K, Motoyoshi K, Kanazawa I. Guillain-Barré syndrome with IgM antibody to the ganglioside GalNAc-GD1a. J Neuroimmunol. 2001;113:260-7. doi: 10.1016/s0165-5728(00)00451-3.
28. Meyer MF, Hellmich B, Kotterba S, Schatz H. Cytomegalovirus infection in systemic necrotizing vasculitis: causative agent or opportunistic infection? Rheumatol Int. 2000;20:35-8. doi: 10.1007/s002960000063.
29. Broccolo F, Iuliano R, Careddu AM, et al. Detection of lymphotropic herpesvirus DNA by polymerase chain reaction in cerebrospinal fluid of AIDS patients with neurological disease. Acta Virol. 2000;44:137-43.
30. Ogawara K, Kuwabara S, Mori M, Hattori T, Koga M, Yuki N. Axonal Guillain-Barré syndrome: relation to anti-ganglioside antibodies and Campylobacter jejuni infection in Japan. Ann Neurol. 2000;48:624-31.
31. Vanpee D, Laloux P, Gillet JB, Esselinckx W. Viral brachial neuritis in emergency medicine. J Emerg Med. 2000;18:177-9. doi: 10.1016/s0736-4679(99)00189-4.
32. de Maar EF, Kas-Deelen DM, de Jager AE, The H, Tegzess AM, van Son WJ. Inflammatory demyelinating polyneuropathy in a kidney transplant patient with cytomegalovirus infection. Nephrol Dial Transplant. 1999;14:2228-30. doi: 10.1093/ndt/14.9.2228.
33. Yuki N, Yamamoto T, Hirata K. Correlation between cytomegalovirus infection and IgM anti-MAG/SGPG antibody-associated neuropathy. Ann Neurol. 1998;44:408-10. doi: 10.1002/ana.410440321.
34. Harada H, Tamaoka A, Yoshida H, et al. Horner's syndrome associated with mononeuritis multiplex due to cytomegalovirus as the initial manifestation in a patient with AIDS. J Neurol Sci. 1998;154:91-3. doi: 10.1016/s0022-510x(97)00204-9.
35. Calore EE, Shulte G, Penalva De Oliveira AC, Cavaliere MJ, Perez Calore NM, Weg R. Nerve biopsy in patients with AIDS. Pathologica. 1998;90:31-5.
36. Corral I, Quereda C, Casado JL, et al. Acute polyradiculopathies in HIV-infected patients. J Neurol. 1997;244:499-504. doi: 10.1007/s004150050132.
37. Irie S, Saito T, Nakamura K, et al. Association of anti-GM2 antibodies in Guillain-Barré syndrome with acute cytomegalovirus infection. J Neuroimmunol. 1996;68:19-26. doi: 10.1016/0165-5728(96)00059-8.
38. Miller RF, Fox JD, Thomas P, et al. Acute lumbosacral polyradiculopathy due to cytomegalovirus in advanced HIV disease: CSF findings in 17 patients. J Neurol Neurosurg Psychiatry. 1996;61:456-60. doi: 10.1136/jnnp.61.5.456.
39. Visser LH, van der Meché FG, Meulstee J, et al. Cytomegalovirus infection and Guillain-Barré syndrome: the clinical, electrophysiologic, and prognostic features. Dutch Guillain-Barré Study Group. Neurology. 1996;47:668-73. doi: 10.1212/wnl.47.3.668.
40. Ben-Smith A, Gaston JS, Barber PC, Winer JB. Isolation and characterisation of T lymphocytes from sural nerve biopsies in patients with Guillain-Barré syndrome and chronic inflammatory demyelinating polyneuropathy. J Neurol Neurosurg Psychiatry. 1996;61:362-8. doi: 10.1136/jnnp.61.4.362.
41. Fox JD, Brink NS, Zuckerman MA, et al. Detection of herpesvirus DNA by nested polymerase chain reaction in cerebrospinal fluid of human immunodeficiency virus-infected persons with neurologic disease: a prospective evaluation. J Infect Dis. 1995;172:1087-90. doi: 10.1093/infdis/172.4.1087.
42. Kuller JA, Katz VL, McCoy MC, Hansen WF. Pregnancy complicated by Guillain-Barré syndrome. South Med J. 1995;88:987-9. doi: 10.1097/00007611-199509000-00022.
43. So YT, Olney RK. Acute lumbosacral polyradiculopathy in acquired immunodeficiency syndrome: experience in 23 patients. Ann Neurol. 1994;35:53-8. doi: 10.1002/ana.410350109.
44. Roullet E, Assuerus V, Gozlan J, et al. Cytomegalovirus multifocal neuropathy in AIDS: analysis of 15 consecutive cases. Neurology. 1994;44:2174-82. doi: 10.1212/wnl.44.11.2174.
45. Mastroianni CM, Sebastiani G, Folgori F, Ajassa C, Vullo V, Volpi A. Detection of cytomegalovirus-matrix protein (pp65) in leukocytes of HIV-infected patients with painful peripheral neuropathy. J Med Virol. 1994;44:172-5. doi: 10.1002/jmv.1890440210.
46. Morgello S, Simpson DM. Multifocal cytomegalovirus demyelinative polyneuropathy associated with AIDS. Muscle Nerve. 1994;17:176-82. doi: 10.1002/mus.880170208.
47. Kim YS, Hollander H. Polyradiculopathy due to cytomegalovirus: report of two cases in which improvement occurred after prolonged therapy and review of the literature. Clin Infect Dis. 1993;17:32-7. doi: 10.1093/clinids/17.1.32.
48. Fuller GN, Jacobs JM, Guiloff RJ. Nature and incidence of peripheral nerve syndromes in HIV infection. J Neurol Neurosurg Psychiatry. 1993;56:372-81. doi: 10.1136/jnnp.56.4.372.
49. Fiala M, Singer EJ, Graves MC, et al. AIDS dementia complex complicated by cytomegalovirus encephalopathy. J Neurol. 1993;240:223-31. doi: 10.1007/BF00818709.
50. Gozlan J, Salord JM, Roullet E, et al. Rapid detection of cytomegalovirus DNA in cerebrospinal fluid of AIDS patients with neurologic disorders. J Infect Dis. 1992;166:1416-21. doi: 10.1093/infdis/166.6.1416. Erratum in: J Infect Dis 1993;167:995.
51. McCormick JN, Wojtacha D, Edmond E. Detection of cytomegalovirus antigens in phagocytosed serum complexes from a patient with rheumatoid arthritis, vasculitis, peripheral neuropathy, cutaneous ulceration, and digital gangrene. Ann Rheum Dis. 1992;51:553-5. doi: 10.1136/ard.51.4.553.
52. Beydoun SR. Misdiagnosis of cytomegalovirus polyradiculopathy, coexisting with HIV neuropathy. Muscle Nerve. 1991;14:575-6.
53. Said G, Lacroix C, Chemouilli P, et al. Cytomegalovirus neuropathy in acquired immunodeficiency syndrome: a clinical and pathological study. Ann Neurol. 1991;29:139-46. doi: 10.1002/ana.410290205.
54. Grafe MR, Wiley CA. Spinal cord and peripheral nerve pathology in AIDS: the roles of cytomegalovirus and human immunodeficiency virus. Ann Neurol. 1989;25:561-6. doi: 10.1002/ana.410250606.
55. Robert ME, Geraghty JJ 3rd, Miles SA, Cornford ME, Vinters HV. Severe neuropathy in a patient with acquired immune deficiency syndrome (AIDS). Evidence for widespread cytomegalovirus infection of peripheral nerve and human immunodeficiency virus-like immunoreactivity of anterior horn cells. Acta Neuropathol. 1989;79:255-61. doi: 10.1007/BF00294659.
56. Winer JB, Hughes RA, Anderson MJ, Jones DM, Kangro H, Watkins RP. A prospective study of acute idiopathic neuropathy. II. Antecedent events. J Neurol Neurosurg Psychiatry. 1988;51:613-8. doi: 10.1136/jnnp.51.5.613.
57. Snider WD, Simpson DM, Nielsen S, Gold JW, Metroka CE, Posner JB. Neurological complications of acquired immune deficiency syndrome: analysis of 50 patients. Ann Neurol. 1983;14:403-18. doi: 10.1002/ana.410140404.
58. Crisp DE, Bray PF, Bloomer LC. Recurrent polyneuropathy with multiple herpesvirus infections. Pediatrics. 1983;71:163-5.
59. Doherty M, Bradfield JW. Polyarteritis nodosa associated with acute cytomegalovirus infection. Ann Rheum Dis. 1981;40:419-21. doi: 10.1136/ard.40.4.419.
